# Effects of Phenolic-Rich Onion (*Allium cepa* L.) Extract on the Growth Performance, Behavior, Intestinal Histology, Amino Acid Digestibility, Antioxidant Activity, and the Immune Status of Broiler Chickens

**DOI:** 10.3389/fvets.2020.582612

**Published:** 2020-11-12

**Authors:** Anaam E. Omar, Hanan S. Al-Khalaifah, Wafaa A. M. Mohamed, Heba S. A. Gharib, Ali Osman, Naif A. Al-Gabri, Shimaa A. Amer

**Affiliations:** ^1^Department of Nutrition and Clinical Nutrition, Faculty of Veterinary Medicine, Zagazig University, Zagazig, Egypt; ^2^Environment and Life Sciences Research Center, Kuwait Institute for Scientific Research, Kuwait City, Kuwait; ^3^Department of Clinical Pathology, Faculty of Veterinary Medicine, Zagazig University, Zagazig, Egypt; ^4^Department of Veterinary Public Health, Faculty of Veterinary Medicine, Zagazig University, Zagazig, Egypt; ^5^Biochemistry Department, Faculty of Agriculture, Zagazig University, Zagazig, Egypt; ^6^Pathology Department, Faculty of Veterinary Medicine, Thamar University, Dhamar, Yemen; ^7^Laboratory of Regional Djibouti Livestock Quarantine, Abu Yaser International Est., Djibouti, Djibouti

**Keywords:** broiler—chicken, onion extract, growth performance, behavior, immunity, gut histomorphology

## Abstract

The effect of phenolic-rich onion extract (PROE), as a feed additive, was evaluated on the growth, carcass traits, behavior, welfare, intestinal histology, amino acid ileal digestibility “AID%,” and the immune status of broiler chicks for 35 days. A total number of 400, 1-day-old broiler chicks (45.38 g ± 1.35) were allocated to four different treatments with 10 replicates each (100 chicks/treatment) consisting of: T1, basal diet without additives (control treatment) (PROE0); T2, basal diet + phenolic-rich onion extract (1 g/kg diet) (PROE1); T3, basal diet + phenolic-rich onion extract (2 g/kg diet) (PROE2); and T4, basal diet + phenolic-rich onion extract (3 g/kg diet) (PROE3). An increase in the final body weight “FBW,” bodyweight gain “BWG,” and feed consumption was observed (*P* < 0.05) at different PROE levels. Also, the thymus and bursa percentages were increased in the PROE2 and PROE3 treatments (*P* < 0.05). The chicks fed on PROE supplemented diets had increased frequency of feeding and drinking and showed comfortable behavior (*P* < 0.05) with lesser aggression (*P* < 0.05). Additionally, an increase was observed in the antioxidant enzyme activity, phagocytic %, phagocytic index, and serum lysozyme activity in PROE supplemented treatments, with the best outcome reported in the PROE3 treatment (*P* < 0.01). IgM was increased in the birds fed with PROE2 and PROE3 diets (*P* < 0.01). PROE supplementation increased the AID% of lysine and methionine (*P* <0.01), PROE3 treatment increased the AID% of threonine (*P* < 0.05), and PROE2 and PROE3 treatments increased the AID% of leucine and isoleucine (*P* < 0.05). Besides, PROE2, and PROE3 treatments increased the villus height and width, mucosal thickness, and goblet cell count from the duodena, jejuna, and ilea (*P* < 0.05) compared to control treatment. Based on these results, we concluded that the dietary addition of phenolic-rich onion extracts can improve the growth rate of broiler chicken by improving the AID% of amino acids and intestinal histology. Also, it can improve the welfare, antioxidant enzymes activity, and immune status of the birds. Phenolic-rich onion extracts can be used as a natural growth promoter in the poultry feed for good health and improved performance.

## Introduction

Feed additives are widely used in the poultry industry to support animal production traits and maintain their good health (1–8). Sub-therapeutic uses of antibiotics as growth promoters in poultry feed have caused controversies, such as drug resistance and residues in the meat (9). These side effects restricted their use in many countries, and on January 1, 2006, antibiotics were strictly prohibited in the poultry feed by the European Union. As a result, efforts are made to find other alternatives to improve broiler chickens' performance and increase their immunity. One of these alternatives was to use onion extract as a promoter of natural growth in poultry production to enhance productivity, control diseases, and improve their immunity. Onions (*Allium cepa* L.) are bulb vegetables that belong to the Liliaceae family. They are widely cultivated in many large-producing countries, such as India, the USA, and China and also used as a common medicinal plant and human food (10).

Onions have numerous biological activities, i.e., as an antioxidant, antimutagenic, and antibacterial (11–13). They have a high content of lectins (the most abundant proteins) along with amino acids cysteine and methionine (14). Dehydrated onions could significantly reduce the serum cholesterol in experimental mice with hypercholesterolemia (15). Similarly, diets supplemented with onions also reduced the serum cholesterol of male albino rats (16). The hypocholesterolemic effect of onion is due to the reduced cholesterol secretion or improved absorption of high-density lipoprotein (HDL) by the liver (17). Onions are a rich source of flavonoids, polyphenols, glycosides, anthocyanins, allicin, and quercetin (18). They are used as a natural antioxidant to prevent oxidation of meat. This antioxidant activity is due to their capacity to scavenge for free radicals and give out electrons or hydrogen atoms (19). The antioxidant activity has been associated with the total phenol content, which is high in red onions, and the flavonoids, mainly quercetin, which is high in yellow onions (347 mg/kg quercetin) (20). Quercetin has powerful antioxidant activity that can protect against diseases caused by oxidative stress (21, 22). One of the studies (23–25) has shown the positive effect of synthetic quercetin on the egg quality and laying performance of hens. Further, a diet supplemented with quercetin reduced the thiobarbituric acid-reactive substance (TBARS) value in broiler meat (26) and pork pie (27). In ruminant nutrition, onion extract improved the feed intake and had a beneficial effect on the leptin and ghrelin concentrations, total antioxidant capacity, and the lambs performance (28).

Despite these results, there was a lack of data on the effect of phenolic-rich onion extract supplementation on chickens' growth, behavior, intestinal tissues, amino acid digestion, and immunity. Thus, the current study was planned to highlight the effect of using different levels, including 1, 2, or 3 g/kg diet of phenolic-rich onion extracts as a natural feed additive, on the broiler chickens' growth performance, carcass traits, behavior, tonic immobility, intestinal histology, amino acids, amino acids' apparent ileal digestibility%, antioxidant enzymes, and their immunity.

## Materials and Methods

### Preparation and Description of the Phenolic-Rich Onion Extracts

The phenolic-rich onion extract was obtained by the technique (29) described previously (30). Fresh red onions (*Allium cepa* L.) were obtained from a native market of Zagazig City, Egypt. The leaves plus the outer skin of fresh onions were closely secluded, and 100 g of onion bulb was mixed and homogenized in 70% methanol (250 mL); this homogenate was stirred for 2 h and then filtered through Whatman No. 2 filter paper. Methanol was removed from the extract using vacuum in a BüCHI-water bath-B-480 evaporator at 45°C, followed by lyophilization in a freeze-Dryer (Thermo-electron Corporation–Heto power dry LL 300 Freeze dryer). Resulting fractions were denoted as phenolic-rich onion extracts (PROE).

The total phenolic compounds (TPCs) were determined in the PROE (1 mg/mL) as described previously (31), using a reagent of Feline-Ciocalteu (diluted with water 1:10, V/V). The calibration equation for gallic acid was y = 0.001x + 0.0563 (R^2^ = 0.9792), where y and x were the absorbance and concentration of gallic acid in μg/mL, respectively.

Total flavonoids (TFs) were evaluated following the protocol stated previously (32). Quercetin was used to obtain the standard curve (10–500 μg/mL) with total flavonoid contents stated as quercetin equivalent (QE) and calculated based on the calibration curve: y = 0.0012x + 0.008 (R^2^ = 0.944), where y was the absorbance and x was the concentration of quercetin in μg/mL.

### Birds

The experiment was done in the poultry research unit of the veterinary medicine faculty, Zagazig University, Egypt, to show the influence of onion extract as a feed additive on the productive parameters, carcass traits, behavior, small intestine histology, amino acids AID%, antioxidant enzymes, and immune status of broiler chickens. The experimental protocol was approved by the Ethics of the Institutional Animal Care and Use Committee of Zagazig University, Egypt (ZUIACUC−2019), and all animal experiments were performed following the recommendations described in “The Guide for the Care and Use of Laboratory Animals in scientific investigations.”

A total number of 400 Ross-308 broiler chicks, aged 1 day, were obtained from a hatchery (Dakahlia Poultry, Mansoura, Egypt) and used in this experiment. They were weighed on arrival (45.38 ± 1.35 g) and further reared using dehydrated solution and neomycin broad-spectrum antibiotic for 3 days until they attained an average weight of 57.01 ± 1.77 g. Birds were raised in a naturally ventilated open house with sawdust as litter. The room temperature was controlled and regulated thermo-statically by two heaters. Room temperature during the first week was established at 34°C and gradually was reduced by 3°C every week until it reached 24°C. The lighting program for the first week was 24 h a day and then was changed to 16 h of light and 8 h of darkness, within 7–35 days. Standard health and vaccination programs against New Castle and Gumboro diseases were conducted. Chicks were daily observed and checked for any syndromes without the mortalities, during the entire experiment. After the study was completed, the remaining birds were released.

### Experimental Design and Diets

Birds were randomly allocated to four treatments (100 chicks for each treatment), with 10 replicates each. The experimental treatments consisted of: T1, basal diet without additives (control treatment) (PROE0); T2, basal diet + phenolic-rich onion extract (1 g/kg diet) (PROE1); T3, basal diet + phenolic-rich onion extract (2 g/kg diet) (PROE2); and T4, basal diet + phenolic-rich onion extract (3 g/kg diet) (PROE3). The experiment was extended to 35 days with free access to feed and water. These diets were given in the mashed form and formulated according to the Ross Manual Guide (33), as presented in Table 1. Different nutrients (DM, CP, and EE) were determined in the experimental feedstuffs and the diets as described by AOAC (34).

**Table 1:** Proximate and chemical composition of the basal diets (%).

**Ingredient**	**Starter stage (4–10 day)**	**Grower stage (11–23 day)**	**Finisher stage (24–35 day)**
Yellow corn	55.64	59.43	62.43
Soybean meal, 48%	31.9	27.9	23.03
Corn gluten, 60%	5.98	5.57	6.57
Soybean oil	2	3	4
Calcium carbonate	1.3	1.2	1.05
Calcium dibasic phosphate	1.5	1.3	1.3
Common salt	0.15	0.15	0.15
Premix^*^	0.3	0.3	0.3
DL- Methionine, 98%	0.23	0.2	0.18
Lysine, HCl, 78%	0.47	0.42	0.46
Choline	0.07	0.07	0.07
Threonine	0.1	0.1	0.1
Phytase	0.01	0.01	0.01
Antimycotoxin	0.1	0.1	0.1
NaCo3	0.25	0.25	0.25
**Chemical analysis (%)**			
ME kcal/kg diet	3008.93	3104.47	3210.19
CP	23.43	21.52	20.09
Ca	0.97	0.87	0.81
Available P	0.48	0.43	0.41
Lysine	1.44	1.29	1.19
Methionine	0.56	0.51	0.48
Threonine	0.97	0.88	0.81

* *premix per kg of diet: vitamin A, 1 500 IU; vitamin D3, 200 IU; vitamin E, 10 mg; vitamin K3, 0.5 mg; thiamine, 1.8 mg; riboflavin, 3.6 mg; pantothenic cid, 10 mg; folic acid, 0.55 mg; pyridoxine, 3.5 mg; niacin, 35 mg; cobalamin, 0.01 mg; biotin, 0.15 mg; Fe, 80 mg; Cu, 8 mg; Mn, 60 mg; Zn, 40 mg; I, 0.35 mg; Se, 0.15 mg*.

*ME, metabolizable energy; CP, crude protein; EE, ether extract; Ca, calcium; P, phosphorus*.

### Growth Performance

When the birds were 4 days old, their initial weights were recorded individually, and the average body weight from each treatment was determined at 10, 23, and 35 days. The body weight gain at each time interval was calculated as the difference between the final and the initial body weight. The average feed intake per bird in each replicate was estimated as the difference between the amount of given food and the residue left, which was then divided by the number of birds in each replicate.

Average daily gain (ADG) (g/bird/day) = final weight gain-initial weight/(number of birds × number of days).

Average daily feed intake (ADFI) (g/bird/day) = cumulative feed intake/(number of birds × number of days).

The feed conversion ratio was assessed as stated by (35) and was calculated as follows; FCR = feed intake (g)/weight gain (g).

### Behavioral Data

#### Behavioral Observation

The behavioral observations were commenced once the birds attained the age of 2 weeks. Video cameras were installed in the pens using scan sampling techniques, and the behavioral observation was performed as follows: each treatment was observed weekly, twice a day (30 min/each time), for 6 days and 6 h for each week. The behavioral patterns were recorded from 7–9 a.m. to 2–4 p.m. The recorded behavioral patterns were feeding, drinking, foraging, sitting, walking, standing, feather preening, and other comfort behaviors, including wing/leg stretching and/or wing flapping, head shaking or body shaking, and aggression. A comprehensive explanation for those patterns was presented previously (36).

For each examination, the number of chicks detected for each behavioral activity was further recorded every 5 min, and results were calculated as the percentage of birds performing the behavior/total observed birds (37).

### Tonic Immobility (TI)

For each treatment, 15 birds were examined for tonic immobility on the 33rd and 34th day (38). TI was forced by putting the broiler on its back with its head hanging in a wooden cradle, resembling the letter U (39), and the broiler was gently reined for 10 s. The examiner took a seat at a distance of about 1 m from the bird without doing unnecessary noise and movements and commenced a stopwatch to register the latencies until the bird adjusted him/herself after the removal of restraint by hand. If they corrected their position in < 10 s, the restraining step was repeated, since there was no induction of tonic immobility. If TI was not achieved after three trials, the time of TI was recorded as 0 s. On the contrary, if the chick failed to correct him/herself after 600 s, the test was ended, and the TI duration was recorded as 600 s (3, 40).

### Carcass Traits

On day 35, ten birds from each treatment, with an average body weight that corresponded to the respective treatment, were selected for the carcass evaluation. They were allowed to fast for 12 h and then weighed, slaughtered until they completely bled out, de-feathered, eviscerated, and finally weighed to determine the dressing percentage. The weights of the dressed carcass, viscera, intestine, liver, heart, and spleen were determined and expressed as a percentage of live body weight.

### Amino Acids Ileal Digestibility

In order to determine the amino acids ileal digestibility, titanium dioxide was used, which is an indigestible indicator substance that does not affect the digestibility of nutrients and has a recovery rate of almost 100%. It was added to the feed at 0.5% dosage (5 kg/t of feed) and used for 5 days. Every assay diet was offered freely to four replicates (five chickens per replicate) between 35 and 40 days of their age. All birds were slaughtered at the end of the experiment, and the contents of the lower half of the ileum were collected in a plastic vessel by gentle flushing with distilled water. Ileal digesta of the chickens within a pen was collected, assembled, and dried by freezing. Dried ileal digesta samples were ground and passed through a 0.5 mm sieve and stored in airtight vessels at −20°C, until further chemical analysis.

The amino acid concentration in the diet and ileal digesta samples were assessed according to Li et al. (41) and Siriwan et al. (42). Tryptophan was determined separately according to Ravindran and Bryden (43). Titanium dioxide was estimated following the procedures described by Fenton and Fenton (44). AID% of amino acids was estimated by the following equation: AID (%) = 100—[(Ti _(diet)_ × AA _(ileum))_/(TI _(ileum)_ × AA _(diet)_) × 100].

Where Ti (diet) was the concentration of titanium dioxide in the diet, Ti (ileal) was the concentration of titanium dioxide in ileal digesta, AA (ileal) was the concentration of the test AA in ileal digesta sample, and AA (diet) was the concentration of the test AA in the diet.

### Sample Collection

At the end of the experiment, birds were made to fast for 12 h and then euthanized by cervical dislocation (45); later blood samples were collected from five birds randomly selected from each experimental treatment. The first sample was collected for hematological analysis (Leukogram) in an EDTA tube; the second sample was collected in heparinized tubes for phagocytosis; meanwhile, the third sample was drawn into a clean, dry centrifuge tube without anticoagulant, which was left to clot at room temperature and then centrifuged for 5 min at 3000 rpm to separate the serum for further clinicobiochemical analysis, including the antioxidant defense system, and selective immunological parameters, such as IgM and lysozyme concentrations. Samples were taken from different parts of the gut for histological examination.

### Hematological Studies

The leukocytic count for chicken's blood was done using an improved Neubauer hemocytometer and a special diluent, namely, Natt and Herrick solution (46). To count the differential leukocytes and detect the abnormalities in RBCs morphology, blood films were prepared, fixed by methyl alcohol, and then stained with Giemsa stain as described previously (47).

### Clinicobiochemical Analysis

Antioxidant defense systems, such as serum levels of CAT and SOD were measured, as stated by Aebi (48) and Nishikimi et al. (49), respectively. The serum level of reduced glutathione (GSH) was measured by the method of Beutler (50). The concentration of serum lysozyme was determined according to Lie et al. (51). The concentration of IgM was determined using chicken ELISA kits of ABCAM Co. with CAT.NO. AB157692, according to the manufacturer's instruction.

### Phagocytic Activity

Phagocytic activity was measured by separating white blood cells (WBCs) from peripheral blood using Ficoll–Histopaque density gradient centrifugation, as described by Hampton et al. (52). We used heat-inactivated *Candida albicans* (*C. Albicans*) in 24-well gelatin/plasma-coated plates to determine the phagocytic capacity of leukocytes, according to the method of Elmowalid (53). A minimum of three slides/bird was assessed, and the cells containing (>10, >20, and >30) FITC-labeled yeast in at least 20 microscopic fields (containing at least 200 cells) were calculated. *C. Albicans* numbers/100 phagocytes were evaluated to yield the phagocytic index of each bird.

### Histopathological Examination of the Small Intestine

The specimens from the intestine were stored in 10% neutral buffered formalin (fixation) and managed until further histological analysis. The specimens were dehydrated with an ascending grade of ethanol (75–100%), then treated with xylol I, II, and later embedded in paraffin and finally sliced into 4 μm longitudinal and cross-sections using a microtome (Leica RM 2155, England). Slides were stained using hematoxylin and eosin by following the method of Bancroft et al. (54). Camera microscope AmScope® software (AmScope digital camera-attached Ceti England microscope) was used for morphometric analysis as follows: villus height was measured (μm) from the tip to the base of villus and diameter. Also, muscular thickness, the thickness of the submucosa layer, the goblet cell numbers per area of the epithelium layer, and the intraepithelial leucocytes were considered as well.

### Statistical Analysis

Data were analyzed with a one-way analysis of variance (ANOVA) using the GLM procedure in SPSS (SPSS Inc., Chicago, IL, USA) after Shapiro-Wilk's test was used to verify the normality and Levene's test was used to verify homogeneity of variance components between experimental treatments. The replicate, or the individual bird, served as an experimental unit for all statistical analyses. The significant difference between the mean values was tested using Duncan's multiple range test (55), and the variation in the data was expressed as pooled SEM, and the significance level was set at *P* < 0.05.

## Results

### Description of the Phenolic-Rich Onion Extract

Onion bulbs contain a high percentage of TPCs. The total phenolic compounds in the onion were 70.55 mg GAE g-1 DW with flavonoids being the main series of these phenolic compounds, representing a high proportion of TPCs in onion (11.8 mg QE g^−1^ DW) (data not shown).

### Growth Performance

The growth performance of Broilers was presented in Table 2. During the starter period, different levels of PROE could increase (*P* < 0.05) the BW, ADG, and ADFI with no effect on FCR when compared to PROE0 treatment. Throughout the grower period, broilers fed with PROE2, and PROE3 diets showed an increase (*P* < 0.05) in the BW and ADG, while broilers fed on the PROE1 diet showed no significant difference compared to the PROE0 treatment. PROE supplemented treatments significantly improved the ADFI and FCR (*P* < 0.05). The finisher period and overall performance results showed that different levels of PROE increased (*P* < 0.05), the BW, ADG, and ADFI. Also, during the finisher period, the FCR was decreased (*P* = 0.04) in PROE1 and PROE2 treatments; however, the overall FCR was not significantly affected (*P* = 0.31). The final body weight was highest in the PROE3 treatment, while the least final body weight was observed in the PROE0 treatment group.

**Table 2:** The effect of PROE supplementation on the growth performance parameters of broiler chickens.

**Item**	**PROE0**	**PROE1**	**PROE2**	**PROE3**	**SEM**	***P-value***
**Initial wt. (g)**	57.01	58.05	58.06	58.61	0.770	0.302
**Starter Period (4–10 day)**						
BW (g)	181.13^b^	218.06^a^	222.50^a^	221.11^a^	5.430	0.001
ADG (g/bird/d)	17.73^b^	22.85^a^	23.49^a^	23.21^a^	0.731	0.001
ADFI (g/bird/d)	26.89^b^	33.08^a^	33.51^a^	32.82^a^	0.872	0.002
FCR	1.52	1.45	1.43	1.42	0.045	0.195
**Grower Period (11–23 day)**						
BW (g)	718.89^b^	754.44^b^	806.11^a^	797.22^a^	17.623	0.001
ADG (g/bird/d)	41.36^b^	41.26^b^	44.89^a^	44.31^a^	0.599	0.015
ADFI (g/bird/d)	64.25^b^	72.78^a^	73.49^a^	73.07^a^	1.447	0.033
FCR	1.55^b^	1.77^a^	1.64^a,b^	1.65^a,b^	0.055	0.074
**Finisher Period (24–35 day)**						
BW (g)	1586.22^b^	1868.03^a^	1870.43^a^	1906.27^a^	40.531	0.003
ADG (g/bird/d)	72.27^b^	92.79^a^	88.69^a^	92.42^a^	2.671	0.002
ADFI (g/bird/d)	140.97^b^	152.68^a,b^	150.74^a,b^	166.43^a^	3.375	0.025
FCR	1.95^a^	1.64^b^	1.70^b^	1.80^a,b^	0.050	0.055
**Overall performance**						
FBW(g)	1586.22^b^	1868.03^a^	1870.43^a^	1906.27^a^	40.532	0.001
ADG (g/bird/d)	47.78^b^	56.56^a^	56.63^a^	57.73^a^	3.564	0.001
ADFI (g/bird/d)	84.85^b^	94.06^a^	93.72^a^	99.28^a^	4.696	0.029
FCR	1.78	1.66	1.65	1.72	0.039	0.318

*Means within the same row carrying different superscripts are significantly different at (P < 0.05)*.

*PROE0, basal diet without additives; PROE1, basal diet + phenolic rich onion extract (1 g/kg diet); PROE2, basal diet + phenolic rich onion extract (2 g/kg diet); PROE3, basal diet + phenolic rich onion extract (3 g/kg diet)*.

*BW, body weight; ADG, Average daily gain; ADFI, Average daily feed intake; FCR, feed conversion ratio*.

*SEM, standard error of mean*.

### Behavioral Data

The impact of PROE supplementation on the broiler's behavior is presented in Table 3. The frequency of ingestive behavior (feeding and drinking) was significantly increased by PROE supplementation. However, the impact of PROE supplementation was not significant on other behaviors, including foraging, sitting, walking, and standing compared to the control treatment. Feather preening and other comfort behaviors were improved (*P* < 0.05) in PROE-supplemented treatments, while abnormal behavior (feather pecking and aggression) was increased (*P* < 0.05) in the PROE0 control. Regarding the tonic immobility (TI), TI duration was not significantly decreased (*P* > 0.05) with PROE supplementation, and TI attempts were not significantly different between the treatments (*P* > 0.05).

**Table 3:** The effect of PROE supplementation on various behaviors (% means of broilers/5 min) and tonic immobility of broiler chickens.

**Behavioral patterns**	**PROE0**	**PROE1**	**PROE2**	**PROE3**	**SEM**	***P-*Value**
Feeding	28.39^b^	39.19^a,b^	46.98^a^	51.79^a^	10.623	0.016
Drinking	16.09^b^	25.65^a^	28.58^a^	28.84^a^	9.512	0.085
Foraging	21.45	19.40	17.37	15.43	3.550	0.645
Resting	59.57	62.49	68.05	71.66	3.044	0.529
Standing	25.65	22.78	18.85	22.54	10.232	0.609
Walking	24.56	23.56	18.10	20.33	8.225	0.415
Feather preening	25.59^b^	27.72^a,b^	21.07^b^	34.76^a^	6.914	0.077
Other comfort^*^	11.07^b^	16.97^b^	18.85^a,b^	28.46^a^	4.581	0.019
Aggression	2.31^a^	0.68^b^	0.36^b^	0.00^b^	0.750	0.038
**Tonic immobility**						
TI attempts^**^	2.16	2.37	2.33	2.66	0.041	0.890
TI duration (sec)	122.33	100.5	84.83	72.16	11.635	0.410

*Means within the same row carrying different superscripts are significantly different at (P < 0.05)*.

* *others comfort behavior included: wing flapping, wing/Leg stretch, body shaking, and head shaking*.

** *TI, Tonic immobility*.

*PROE0, basal diet without additives; PROE1, basal diet + phenolic rich onion extract (1 g/kg diet); PROE2, basal diet + phenolic rich onion extract (2 g/kg diet); PROE3, basal diet + phenolic rich onion extract (3 g/kg diet)*.

### Carcass Traits

The effect of PROE supplementation on the carcass trait percentage is shown in Table 4. Dressing percentage was not affected (*P* > 0.05), while the percentage of thymus and bursa was increased (*P* < 0.05) in PROE2 and PROE3 treatments but was not significantly (*P* > 0.05) increased in the PROE1 treatment compared to the PROE0 control. Also, no significant (*P* > 0.05) changes in the percentage of the intestine, gizzard, liver, spleen, and heart were observed between PROE supplemented and the PROE0 treatment.

**Table 4:** The effect of PROE supplementation on the carcass traits percentages relative to the live body weight.

**Item**	**PROE0**	**PROE1**	**PROE2**	**PROE3**	**SEM**	***P-value***
Intestine	5.14	6.36	6.05	6.49	0.171	0.335
Spleen	0.09	0.08	0.09	0.09	0.009	0.881
Bursa	0.09^b^	0.14^a,b^	0.17^a^	0.18^a^	0.022	0.026
Thymus	0.33^b^	0.41^a,b^	0.48^a^	0.53^a^	0.035	0.018
Gizzard	2.47^a,b^	2.97^a^	2.79^a,b^	2.35^b^	0.081	0.074
Liver	2.02	2.10	2.21	1.92	0.077	0.708
Carcass	64.81	63.46	63.63	64.02	0.425	0.725
Heart	0.41	0.44	0.39	0.39	0.023	0.841

*Means within the same row carrying different superscripts are significantly different at (P < 0.05)*.

*PROE0, basal diet without additives; PROE1, basal diet + phenolic rich onion extract (1 g/kg diet); PROE2, basal diet + phenolic rich onion extract (2 g/kg diet); PROE3, basal diet + phenolic rich onion extract (3 g/kg diet)*.

### Ileal Digestibility of Amino Acids

The impact of different onion extract levels on the AID% of amino acids is highlighted in Table 5. The results revealed that different levels of PROE supplementation statistically improved (*P* < 0.01) the AID% of lysine and methionine but did not have a significant influence (*P* > 0.05) on the AID% of tryptophan and valine when compared to the PROE0 treatment. The AID% of threonine was improved (*P* < 0.01) in PROE3 treatment, while it did not differ (*P* > 0.05) in PROE1 and PROE2 treatments when compared to the control. Also, a significant improvement in AID% of leucine and isoleucine (*P* < 0.01) and a significant increase in AID% of arginine (*P* = 0.03) was observed in PROE2 and PROE3 diets compared to the PROE0 control.

**Table 5:** The effect of PROE supplementation on the apparent ileal digestibility coefficient (AID%) of amino acids.

**Item**	**PROE0**	**PROE1**	**PROE2**	**PROE3**	**SEM**	***P-value***
Lysine	83.54^c^	83.95^b^	85.00^a^	85.00^a^	0.180	0.001
Methionine	88.40^d^	88.91^c^	89.07^b^	89.83^a^	0.074	0.003
Threonine	85.18^b^	84.44^c^	84.32^c^	85.68^a^	0.115	0.001
Tryptophan	87.02	86.54	86.54	86.71	0.244	0.528
Arginine	89.08^a,b^	88.99^b^	89.25^a^	89.22^a^	0.058	0.035
Valine	84.83	84.91	84.83	84.83	0.077	0.904
Leucine	90.05^c^	90.05^c^	90.21^b^	90.63^a^	0.046	0.004
Isoleucine	84.91^b^	84.79^b^	85.15^a^	85.27^a^	0.093	0.001

*Means within the same row carrying different superscripts are significantly different at (P < 0.05)*.

*PROE0, basal diet without additives; PROE1, basal diet + phenolic rich onion extract (1 g/kg diet); PROE2, basal diet + phenolic rich onion extract (2 g/kg diet); PROE3, basal diet + phenolic rich onion extract (3 g/kg diet)*.

### Serum Antioxidant Activity

The effect of PROE on the serum antioxidant activity of broiler chickens is represented in Table 6. Compared to the control treatment, a significant increase was observed in the CAT, SOD activity, and GSH level in all PROE supplemented treatments (*P* < 0.01).

**Table 6:** The effect of PROE supplementation on the antioxidant activity, selective immunological parameters, and Leukogram of broiler chickens.

**Item**	**PROE0**	**PROE1**	**PROE2**	**PROE3**	**SEM**	***P-Value***
**Antioxidant indices**						
Serum CAT (U/L)	556.67^b^	582.33^a^	593.33^a^	593.67^a^	4.175	0.001
Serum SOD (U/mL)	23.40^c^	29.80^b^	37.90^a^	40.87^a^	2.496	0.001
Serum GSH(mmol/L)	3.40^c^	6.93^b^	8.43^a^	9.03^a^	0.654	0.002
**Immunological parameters**						1.81
Phagocytic%	54.00^d^	60.00^c^	64.00^b^	70.00^a^	3.149	0.004
Phagocytic index	2.73^d^	3.60^c^	4.43^b^	5.23^a^	0.238	0.001
IgM (ng/mL)	38.33^c^	44.00^b,c^	49.00^b^	58.67^a^	1.047	0.001
Lysozyme (mg/L)	1.18^d^	1.46^c^	1.81^b^	2.06^a^	0.054	0.002
**Leukogram**						
TLC (×10^3^/μL)	2.38	2.46	2.62	2.71	0.715	0.715
Neutrophils (×10^3^/μL)	0.23	0.23	0.23	0.22	0.024	0.980
Eosinophils (×10^3^/μL)	0.03	0.04	0.04	0.03	0.002	0.970
Lymphocytes (×10^3^/μL)	1.98	1.97	2.08	2.13	0.361	0.945
Monocytes (×10^3^/μL)	0.12^c^	0.21^b^	0.26^a^	0.31^a^	0.012	0.001

*Means within the same row carrying different superscripts are significantly different at (P < 0.05)*.

*PROE0, basal diet without additives; PROE1, basal diet + phenolic rich onion extract (1 g/kg diet); PROE2, basal diet + phenolic rich onion extract (2 g/kg diet); PROE3, basal diet + phenolic rich onion extract (3 g/kg diet)*.

### Immunological Parameters

**Figure 1:**
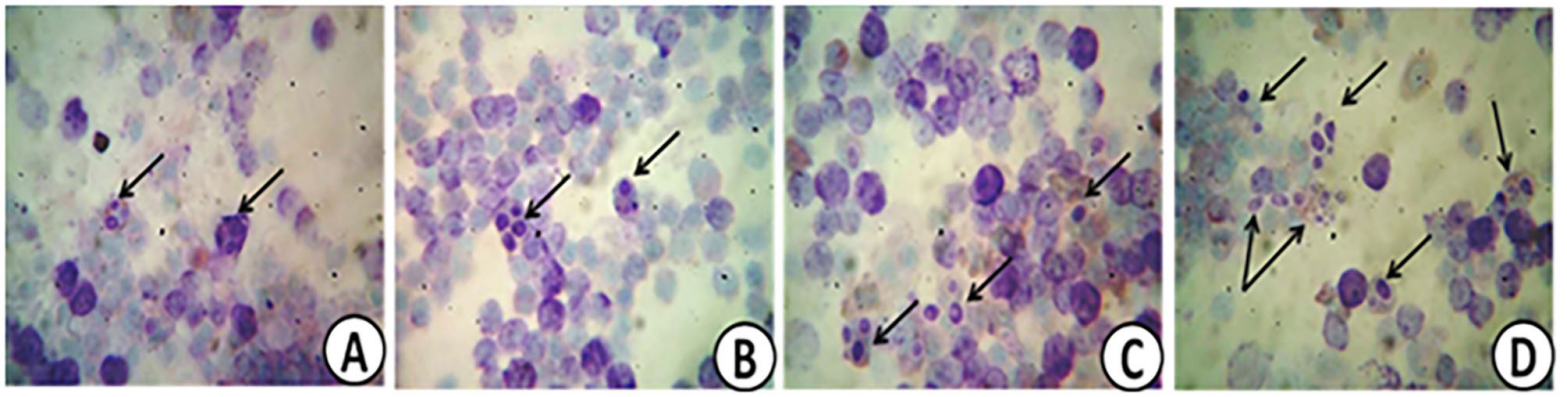
Representative images showing normal **(A)**, mild **(B)**, moderate **(C)**, and marked **(D)** phagocytic activities in PROE0, PROE1, PROE2, and PROE3 treatments, respectively.

The effect of PROE on the selective immunological parameters and leukogram data of broiler chickens is highlighted in Table 6. Different levels of onion extract supplementation increased phagocytic%, phagocytic index, IgM level, and lysozyme activity (*P* < 0.01). The phagocytic activity was normal in the control treatment while it was mild, moderate, and marked in PROE1, PROE2, and PROE3 treatments, respectively (Figure 1). These changes were greater in the PROE3 treatment, followed by the PROE2 treatment. Meanwhile, in all the PROE supplemented treatments, the total leucocytic count (TLC), neutrophils, eosinophil, and lymphocytes were not significantly changed (*P* > 0.05), but the monocytes were increased (*P* < 0.01).

### Histological Finding

**Figure 2:**
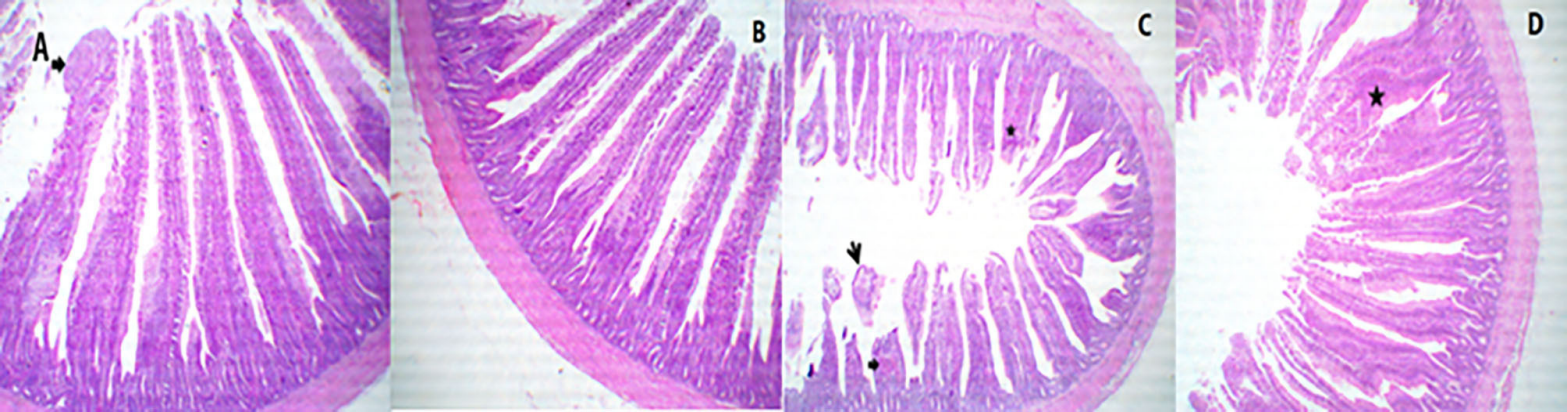
A representative photomicrograph of 40× magnification H&E stained small intestine sections of the broiler chickens. The sections from the duodenal segments from PROE0group showed separate tall and arranged intestinal villi with (arrow) free lumen **(A)**, the duodenal segments from PROE1 group showed apparently separated tall and arranged intestinal villi with free lumen **(B)**, the duodenal segments from PROE2 group revealed marked active intestinal villi and crypts which characterized by limited goblet cells metaplasia (star)' increased sizes and rows of enterocytes (thick arrow) with arranged lamina propria beside some desquamated villi (small arrow) **(C)**. Chicken fed on PROE3 diet revealed marked thin, tall, and separate villi with mild goblet cell proliferations (star) **(D)**.

**Figure 3:**
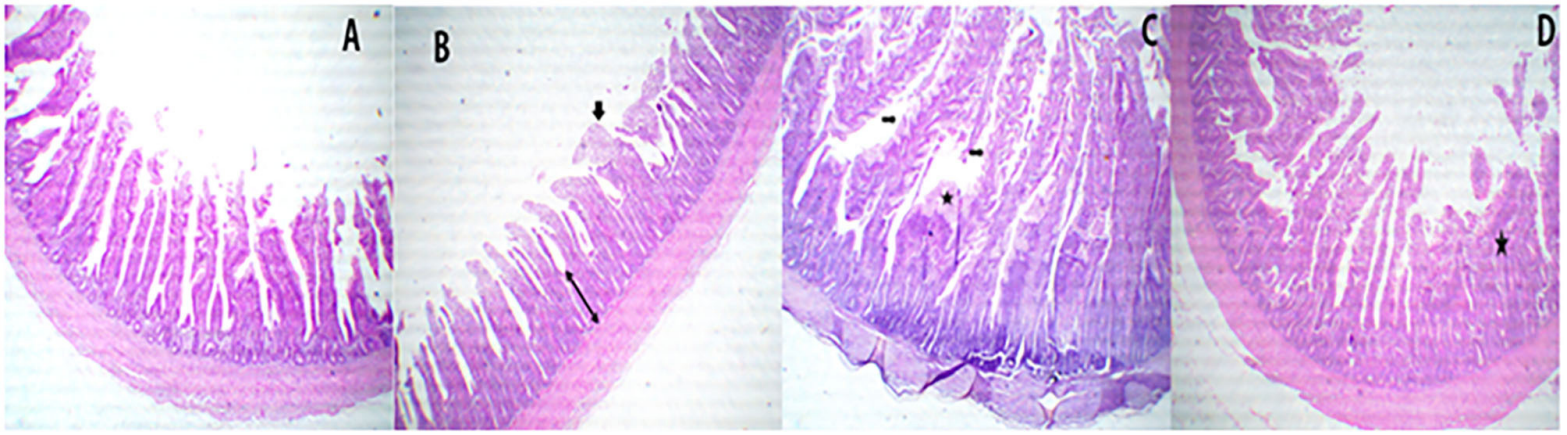
A representative photomicrograph of 40× magnification H&E stained small intestine sections of the broiler chickens. The sections from the jejunal segments of PROE0 group showed free lumen with nearly normal villus structures **(A)**, the sections from the jejunal segments of PROE1 group showed free lumen, a few denuded villi tips with increase intestinal crypt layer depth (towheads arrow) besides partial fusion (arrow) **(B)**, and the jejunal segments from PROE2 group showed separate tall villi and marked serrated surfaces (arrows) with goblet cell metaplasia (star) beside partial fusion some villus **(C)**. In addition to, increased intestinal glands layer besides partial destructed villi with some fusion's villi (star) were observed in PROE3 group **(D)**.

**Figure 4:**
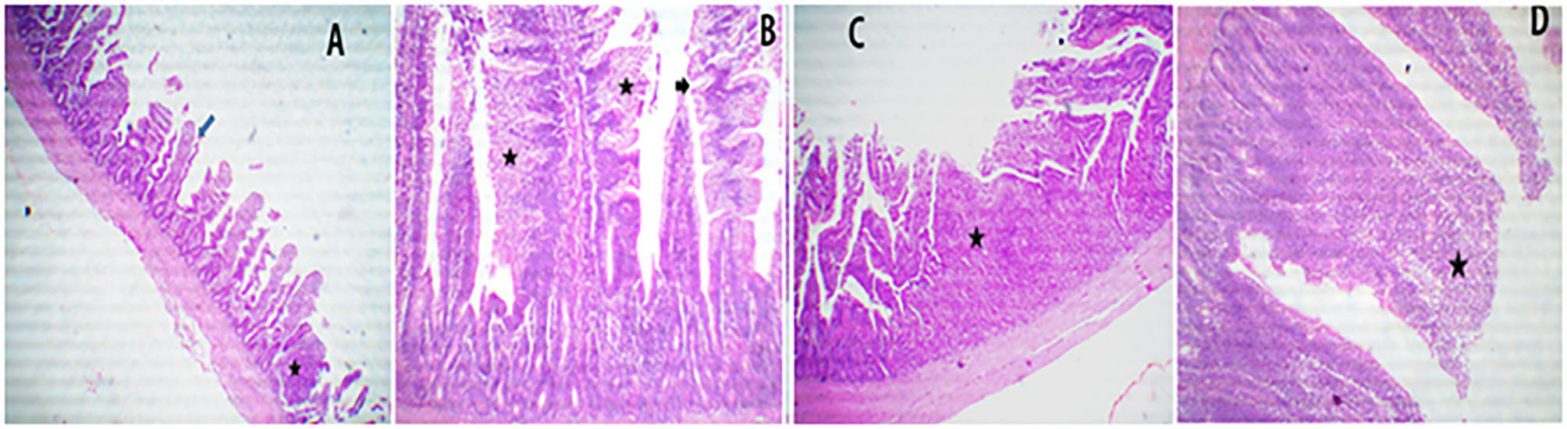
A representative photomicrograph of 40× magnification H&E stained small intestine sections of the broiler chickens. The sections from the ileal segments of PROE0 group showed free lumen and nearly normal limit gut associated lymphoid follicles (star) with a few denuded villi (arrow) **(A)**, the sections from ileal segments of PROE1 group showed thickened and serrated (arrow) of villi due to enterocytes and goblet cell metaplasia (stars) with narrowing of the lamina propria **(B)**. The sections from PROE2 group showed marked hyperplastic intestinal gut associated lymphoid follicles (star) **(C)**. The sections from PROE3 showed marked fusion villi due to increase enterocytes proliferations and goblet cells metaplasia (star) **(D)**.

A representative photomicrograph of H&E-stained small intestine sections of broiler chickens in 40 × magnification is shown in Figures 2–4. Sections of the duodenal segments from PROE0 to PROE1 treatment showed separated tall and arranged intestinal villi with a free lumen (Figures 2A,B), whereas duodenal segments from PROE2 treatment revealed markedly active intestinal villi and crypts, characterized by limited goblet cells metaplasia, increased sizes and rows of enterocytes with arranged lamina propria besides some desquamated villi (Figure 2C) while the chickens fed with the PROE3 diet revealed markedly thin, tall, and separate villi with mild goblet cell proliferation (Figure 2D).

Sections from the jejunal segments of PROE0 treatment showed free lumen with nearly normal villus structures (Figure 3A), while sections of the jejunal segments from the PROE1 treatment group showed free lumen and a few denuded villi tips with increased intestinal crypt layer depth besides partial fusion (Figure 3B). The jejunal segments from PROE2 treatment showed separate tall villi and markedly serrated surfaces with goblet cell metaplasia besides a partial fusion of some villus (Figure 3C), whereas in the PROE3 treatment, an increase in intestinal gland layer was observed besides partially destructed villi with some fused villi (Figure 3D).

Sections of the ileal segments from PROE0 treatment showed free lumen and nearly a normal limit gut-associated lymphoid follicles with a few denuded villi (Figure 4A), while the ileal sections from the PROE1 treatment showed thickened and serrated villi due to the enterocytes and goblet cell metaplasia along with narrowing of the lamina propria (Figure 4B), the sections from the PROE2 treatment showed markedly hyperplastic intestinal gut-associated lymphoid follicles (Figure 4C), and the sections from the PROE3 group showed marked fusion villi due to an increase in enterocytes proliferation and goblet cell metaplasia (Figure 4D).

### Small Intestine Morphometric Measures

The results of morphometric intestinal measures are shown in Table 7. Birds fed with PROE2 and PROE3 diets showed (*P* < 0.05) an increase in the villus height and width, mucosal thickness, and goblet cell numbers in different sections of the small intestine, while different levels of onion extract supplementation significantly increased the duodenal and ileal crypt depth. In the PROE3 treatment group, an improvement (*P* < 0.05) was observed in the duodenal, jejunal, and ileal intra-epithelium lymphocytic linked-cells infiltration and the ileal crypt depth, compared to PROE0 control.

**Table 7:** The effect of PROE supplementation on the morphometric measures of the small intestine of broiler chickens.

**Item**	**PROE0**	**PROE1**	**PROE2**	**PROE3**	**SEM**	***P-Value***
**Duodenum**						
Villous height	966.10^b^	966.10^b^	1366.10^a^	1399.44^a^	70.750	0.002
Villous width	187.19^c^	203.86^b,c^	270.53^a,b^	337.19^a^	27.194	0.001
Crypt depth	120.70^c^	187.37^b^	220.70^a^	220.70^a^	47.771	0.001
Mucosal depth	137.37^c^	150.70^b,c^	197.37^a,b^	237.37^a^	22.484	0.004
Goblet cells count	25.00^b^	29.33^b^	49.33^a^	52.67^a^	6.223	0.028
IELI	175.67^b^	309.00^a,b^	375.67^a,b^	509.00^a^	27.594	0.025
**Jejunum**						
Villous height	1127.63^b^	1127.63^b^	1427.63^a^	1560.96^a^	39.961	0.001
Villous width	171.30^b^	211.30^b^	264.64^a^	277.97^a^	21.712	0.004
Crypt depth	115.29^c^	171.96^b^	198.62^a,b^	221.96^a^	25.005	0.001
Mucosal depth	188.62^c^	195.29^b,c^	238.62^b^	288.62^a^	14.130	0.001
Goblet cells count	17.33^c^	20.67^c^	27.33^b^	37.33^a^	10.890	0.001
IELI	156.33^b^	173.00^b^	199.67^b^	256.33^a^	28.321	0.003
**Ileum**						
Villous height	937.85^b^	971.19^b^	1404.52^a^	1504.52^a^	35.066	0.001
Villous width	153.48^b^	203.48^a,b^	253.48^a^	253.48^a^	25.505	0.011
Crypt depth	138.78^b^	172.11^a,b^	188.78^a,b^	205.45^a^	17.406	0.071
Mucosal depth	172.11^b^	188.78^b^	242.11^a^	272.11^a^	19.891	0.001
Goblet cells count	26.00^b^	29.33^b^	39.33^a,b^	52.67^a^	6.184	0.082
IELI	323.00^b^	423.00^a,b^	556.33^a,b^	656.33^a^	51.152	0.065

*Means within the same row carrying different superscripts are significantly different at (P < 0.05)*.

*IELI, Intra-epithelium lymphocytic lick cells infiltrations*.

*PROE0, basal diet without additives; PROE1, basal diet + phenolic rich onion extract (1 g/kg diet); PROE2, basal diet + phenolic rich onion extract (2 g/kg diet); PROE3, basal diet + phenolic rich onion extract (3 g/kg diet)*.

## Discussion

Our study assessed the effects of using phenolic-rich onion extract, containing high amounts of TPCs and TFCs, as a natural feed additive on the growth performance, behavior, TI, carcass traits, AID% of amino acids, intestinal tissues, antioxidant enzymes, and immunity of broiler chickens. The total phenolic compounds and total flavonoids are recorded as 70.55 mg GAE g-1 DW and 11.8 mg QE g-1 DW, respectively. The total phenolic compounds were previously recorded in different types of onions (56), which ranged from 4.6 to 74.1 mg/g GAE for red, purple, white, and green onion cultivars (57). Flavonoids' data was also consistent with the other publications on the TPCs (58).

### Growth Performance and Digestibility

Our results showed a positive effect of PROE as a dietary supplement on BW, ADG, and ADFI of broiler chickens, and the best results were with the highest level of supplementation. This overall improvement in the growth criteria may be due to the improvement in birds' health, amino acid digestibility, intestinal health, and an increase in their absorptive surface. Herbal products control the growth of many pathogenic and non-pathogenic microbes in the broiler intestine, increasing the efficiency of feed use, and improving the growth rate (59). Onion extract also contains active compounds, including phenols, polyphenols, terpenoid, polypeptides, lectin, alkalis, and essential oils that stimulate digestion (60, 61) and promote growth. Onion also stimulates the synthesis of bile acid and pancreatic enzyme activity, mainly lipase and amylase, ultimately improving fat digestion (62). Moreover, onions contain non-digestible prebiotic and fructooligosaccharide (FOS) components, which are fermented by bifidobacteria to further maintain intestinal and colon health (63). Another reason for the improved AID ratio of amino acids (26) and increased feed consumption (17, 64) could be the increased retention of the total pathway of energy and ether extract. The increased feed consumption is due to the favorable taste and flavor of onion extract (65). Onion has sulfur-containing compounds, including *S-Methylcysteine sulfoxide* (SMCS) and *S-allyl cysteine sulfoxide* (SACS) that reduces the blood sugar levels and stimulates growth by accelerating the glucose flow into the tissues and increasing the thyroid activity (17).

Goodarzi and Nanekarani (66) assessed the addition of 1 and 2% onion extract in the drinking water of broiler chickens and reported an increased ADFI in 1% onion extract supplementation during the grower and total period. Farahani et al. (63) assessed 1% onion extract supplementation in the drinking water of two strains of broiler chickens (Ross and Cobb) and confirmed the positive effects of onion extract on the growth and blood parameters of both strains.

Aditya et al. (26) demonstrated that BWG is generally higher in broiler chickens fed with 7.5 g/kg of onion extract, and the feed intake was increased by supplementing 5, 7.5, and 10 g of onion extract/kg to the diet. An increase in the body weight, BWG, and feed consumption were observed in both the broiler strains “Cobb and Ross” by supplementing liquid onion extract in the drinking water (63). Goodarzi et al. (67) reported a positive effect of 3% onion supplementation on broiler growth parameters. Aji et al. (64) found that the BW, BWG, and feed intake were higher in chicks that were fed with onions and garlic diets. An et al. (68) reported that chicks fed with 0.3% or 0.5% onion extract-supplemented diets showed a slight increase in final body weight and BWG compared to the non-supplemented ones, with no effect on feed consumption during the start-up and farm stages. However, Al-Homidan (69) observed no effect on the feed intake of the broilers by 2% supplementation of dried onion to the feed but observed a reduced feed intake by supplementing 6% dried onion.

### Behavior and Welfare

Our results showed a significant effect of PROE supplementation on feeding and drinking behavior of broiler chickens, which may be due to an increase in the FI of the supplemented treatment group compared to the non-supplemented one (26, 64). However, Ramamneh (70) found no effect of liquid onions on the behavior of broiler chickens. Our study indicated that comforting behavior (feather preening, wing/Leg stretching, wing flapping, head shaking, or body shaking) was improved by adding onion extract to the basic diet. Also, there was a decrease in the aggression in the treatment groups fed with enriched onion diets compared to the non-supplemented diet. These positive changes in the behavior of the broiler chickens fed with onion extract supplemented diet may be attributed to the fact that onions act as an antioxidant and anti-stress agent.

Duration of TI is a method for assessing the level of fear (71, 72) where longer duration of TI indicates a high level of fear and vice versa (73). A high level of fear can negatively affect the well-being and performance of the birds (74). Our results showed that the duration of TI was non-significantly decreased by adding onion extract to the diet, which may be attributed to a decrease in the stress level of the birds, improving their welfare. These results coincided with Mohamed et al. (75), who stated that supplementing onion and garlic in the diet of birds, provides a feasible way to improve their welfare by mitigating the clinicopathological changes.

### Carcass Traits

Our study revealed the insignificant effect of PROE supplementation on the percentages of carcass dressing, intestine, viscera, liver, spleen, and heart weights. Similarly, in other reports, carcass dressing, abdominal fat percentage, and relative weights of the heart, liver, and spleen were not also affected by supplementing onion extract in broiler chicken meals (26, 68). Also, Aji et al. (64) found no effect on the carcass yield of broiler chickens by supplementing onion and garlic in the feed.

### Antioxidant Activity

Onion extract is a rich source of phenolic compounds and flavonoids such as allicin, quercetin, campherol, caffeic acid, gallic acid, para coumaric acid, vanillic acid, and salicylic acid, all of which have potent antioxidant properties. Our results showed increased serum antioxidant enzymes, CAT, SOD, and GSH as a result of PROE supplementation, which can be attributed to the onion's total phenolic and flavonoid contents that were reported to be 70.55 mg GAE g-1 DW and 11.8 mg QE g-1 DW, respectively. These results are consistent with the results of Chang et al. (76), who revealed the positive effects of onion extract on the antioxidant activity. Aditya et al. (26) reported that onion extract supplementation improved the antioxidant capacity and quality of the meat and attributed these results to the total polyphenol content of onion extract which was recorded as 0.39 g/kg with a quercetin concentration of 0.36 g/kg OE, which represented 92.3% of the total polyphenol.

### Immunological Parameters

In our study, PROE supplementation significantly improved the immune response of birds represented by increased IgM concentration, phagocytic%, and phagocytic index and increasing weights of thymus and bursa fabrics. The excess weight of lymph organs in PROE treatments can be attributed to the active compounds in onions along with the antibacterial, antiviral, antioxidant, and anti-inflammatory activities that stimulate positive effects on these organs (77). The oversized follicles lead to an increased immune globulin synthesis (78). Our results were consistent with Hanieh et al. (79) who reported that humoral immune function was improved by supplementing garlic and onions in the chicken diet, after vaccinating the birds with the Newcastle Virus (NDV), sheep red blood cells (SRBC), and *Brucella abortus* (BA). Also, Goodarzi et al. (17) reported an improvement in follicle and spleen weight by supplementing 30 g/kg of fresh onion in fattening meals. Further, the IgG level in onion extract treatments was also increased against the control treatment (26). Also, no effect was observed on the antibody titer against the Newcastle disease virus by using garlic (80) and onions (17) in broiler diets. The data of the blood parameters were found to be consistent with the result of El-Katcha et al. (81) where dietary supplementation with 25, 50, 75, or 100 mg of allicin/kg did not affect the white blood cell, lymphocytes, neutrophils, eosinophils, and basophil percentages of broiler chickens. However, phagocytosis showed significant improvement.

### Histological Finding

The positive effect of phenolic-rich onion extract on villus height and width, crypt depth, mucous thickness, and goblet cell count of duodenum, jejunum, and ileum may be attributed to the fact that including herbs in poultry diets promotes the development and enzymatic activity of the intestinal structure. Our results were consistent with different researchers who reported positive effects of onion powder on villus height and width, crypt depth, and small intestine absorptive surface (82). Moreover, Mahmood et al. (83) reported that the height of villi, the depth of the crypt, and the surface area of jejuna increased significantly by including onions in broiler chicken feed.

Based on our results, we conclude that using phenolic-rich onion extract as a feed additive in broiler chicken diets can improve their growth performance represented by increased body weight, average daily gain, and average daily feed intake in a dose-dependent manner by improving AID% of amino acids and integrating the intestinal histology. It can also improve the birds' behavior and tonic immobility, along with enhancing the antioxidant activity which is represented by increased CAT, SOD activity, and GSH level and act as an immunomodulatory substance by improving the immune status of birds through an increased IgM level, phagocytic percentage, phagocytic index, and increased weights of thymus and bursa fabrics. Therefore, the PROE could be used as an alternative natural growth promoter, immune stimulant, and an antioxidant for broiler production.

## Data Availability Statement

The datasets generated for this study are available on request to the corresponding author. Requests to access these datasets should be directed to Shimaa A. Amer, shimaa.amer@zu.edu.eg.

## Ethics Statement

The animal study was reviewed and approved by the Ethics of the Institutional Animal Care and Use Committee of Zagazig University, Egypt (ZUIACUC−2019), and all animal experiments were performed following recommendations described in The Guide for the Care and Use of Laboratory Animals in scientific investigations.

## Author Contributions

AOm and SA: design of the experiment. AOm, SA, HA-K, WM, HG, AOs, and NA-G: methodology. AOm, SA, WM, and HG: data collection and analysis. AOm, SA, and HG: writing of the manuscript. All authors have read and approved the manuscript.

## Conflict of Interest

The authors declare that the research was conducted in the absence of any commercial or financial relationships that could be construed as a potential conflict of interest.
